# Alisertib Added to Rituximab and Vincristine Is Synthetic Lethal and Potentially Curative in Mice with Aggressive DLBCL Co-Overexpressing MYC and BCL2

**DOI:** 10.1371/journal.pone.0095184

**Published:** 2014-06-03

**Authors:** Daruka Mahadevan, Carla Morales, Laurence S. Cooke, Ann Manziello, David W. Mount, Daniel O. Persky, Richard I. Fisher, Thomas P. Miller, Wenqing Qi

**Affiliations:** 1 Department of Medicine, The University of Tennessee Health Science Center, Memphis, Tennessee, United States of America; 2 Department of Medicine, The University of Arizona Cancer Center, Tucson, Arizona, United States of America; 3 Department of Medicine, Fox Chase Cancer Center – Temple Health, Philadelphia, Pennsylvania, United States of America; Columbia University, United States of America

## Abstract

Pearson correlation coefficient for expression analysis of the Lymphoma/Leukemia Molecular Profiling Project (LLMPP) demonstrated Aurora A and B are highly correlated with *MYC* in DLBCL and mantle cell lymphoma (MCL), while both Auroras correlate with *BCL2* only in DLBCL. Auroras are up-regulated by *MYC* dysregulation with associated aneuploidy and resistance to microtubule targeted agents such as vincristine. Myc and Bcl2 are differentially expressed in U-2932, TMD-8, OCI-Ly10 and Granta-519, but only U-2932 cells over-express mutated p53. Alisertib [MLN8237 or M], a highly selective small molecule inhibitor of Aurora A kinase, was synergistic with vincristine [VCR] and rituximab [R] for inhibition of cell proliferation, abrogation of cell cycle checkpoints and enhanced apoptosis versus single agent or doublet therapy. A DLBCL (U-2932) mouse model showed tumor growth inhibition (TGI) of ∼10–20% (p = 0.001) for M, VCR and M-VCR respectively, while R alone showed ∼50% TGI (p = 0.001). M-R and VCR-R led to tumor regression [TR], but relapsed 10 days after discontinuing therapy. In contrast, M-VCR-R demonstrated TR with no relapse >40 days after stopping therapy with a Kaplan-Meier survival of 100%. Genes that are modulated by M-VCR-R (CENP-C, Auroras) play a role in centromere-kinetochore function in an attempt to maintain mitosis in the presence of synthetic lethality. Together, our data suggest that the interaction between alisertib plus VCR plus rituximab is synergistic and synthetic lethal in Myc and Bcl-2 co-expressing DLBCL. Alisertib plus vincristine plus rituximab [M-VCR-R] may represent a new strategy for DLBCL therapy.

## Introduction

Chromosomal translocations are diagnostic and pathogenic hallmarks of B-cell lymphomas (B-NHL). Double-hit (DH) B-NHL are defined by a chromosomal breakpoint affecting the *MYC* (8q24) locus most frequently associated with a *BCL2* translocation, t(14;18)(q32;q21) [1], [2]. DH B-NHLs are mostly DLBCL, and can be either ABC or GCB phenotype with associated Bcl2 expression [3]. These patients present with poor prognostic features, including elevated LDH, bone marrow and CNS involvement, and a high IPI score [4]. Compared to *MYC+/BCL2*+DHL, *BCL6+/MYC*+DHL are less common, and most represent *BCL2+/BCL6+/MYC*+ triple-hit lymphomas [1]. Also in aggressive types of mantle cell lymphomas (MCL), *CCND1* [t(11;14)] and *MYC* with involvement of 11q13 are frequent [1]. Hence *MYC* activation may be an important oncogenic pathway in both DH-DLBCL and MCL.

DLBCL associated with *MYC* translocations, with or without *BCL2* translocation are associated with inferior survival with R-CHOP therapy [1]. Two recent articles [5], [6], demonstrated concomitant over-expression of Myc (cut point >40%) and Bcl2 (cut point >50%) protein by immunohistochemistry (IHC) in DLBCL patients treated with R-CHOP [5], [6] was associated with inferior overall and progression-free survival only when Bcl2 protein was co-expressed with Myc (P<0.001) [5], [6]. Since current chemo-immunotherapy regimens are ineffective for patients with DH-DLBCL, novel therapeutic strategies based on Myc and Bcl2 biology are needed.

Therapy for DH-DLBCL is an unmet need with two potential novel agents on the horizon. Targeting Bcl2 with a small-molecule inhibitor (ABT-263) in cell lines with t (14∶18) and t(8;14), sensitizes these double hit cells to conventional therapeutic agents [7]. It is established that aberrant Myc protein expression induces Aurora A and B expression and inhibition Aurora enzyme activity in a mouse model enhances apoptosis in *MYC*-driven DLBCL [8]. Auroras are Ser/Thr kinases that play key roles in mitotic initiation, progression and spindle assembly checkpoint activity during the cell cycle. Alisertib (MLN8237 or M), an investigational specific inhibitor of Aurora A, is under development for aggressive B- [9] and T-NHL [10], and preliminary results suggest clinical activity [11].

Analysis of the LLMPP data showed that Aurora A and B expression is predictive of survival in MCL but not in DLBCL [12]. A mouse xenograft model of MCL (Granta-519) showed tumor regression with MLN8237 [M or alisertib] plus vincristine [VCR] but mice relapsed 20 days after discontinuing therapy. In contrast, M-VCR plus rituximab [MVR] was curative [10]. We hypothesized that mechanistic synergy of mitotic spindle inhibition with M-V in MCL would also be effective in Myc+/Bcl2+DHL. Collectively, our data demonstrates that in Myc and Bcl2 co-expressing B-NHL cells, M-V and M-VCR-R therapies were synergistic in inhibiting cell proliferation with enhanced apoptosis. Gene expression profiling informs that M-VCR-R modulates genes associated with karyokinesis.

## Materials and Methods

All procedures were completed in accordance with the University of Arizona Institutional Animal Care and Use Committee (IACUC). Protocol # 10-178, approved August 4, 2013.

### Cells and Reagents

B-NHL cell lines used in this study (TMD-8, U-2932, OCI-Ly10 and Granta-519) were from the Lymphoma SPORE tissue bank (Dr. L. Rimsza, University of Arizona) and Dr. E. Davis (MD Anderson Cancer Center, TX) and maintained in RPMI 1640 medium (Mediatech, VA) supplemented with 10% fetal bovine serum, 2 mM sodium pyruvate and 100 units/ml penicillin/streptomycin at 37°C in a humidified atmosphere containing 5% CO_2_. MLN8237 was kindly provided by Millennium Pharmaceuticals Inc (Cambridge, MA). Rituximab, and VCR were a kind donation by the Arizona Cancer Center Clinic. The compounds were dissolved at 10 mM in DMSO as a stock solution, and then further diluted to desired concentrations for *in vitro* experiments. Anti-Myc (N-262), Anti-p53 (DO-1), anti-Bcl2 (C-21) and anti-PARP (H-250) antibodies were purchased from Santa Cruz Biotechnology (Santa Cruz, CA). Anti-BTK antibody was obtained from BD Biosciences (San Jose, CA). Anti-Aurora B (ab2254) antibody was purchased from Abcam (Cambridge, MA), and anti-GAPDH (14C10) antibody was from Cell Signaling Technology (Danvers, MA).

### Analysis of the LLMPP for Aurora A, Aurora B, MYC and BCL2 Expression in MCL and DLBCL

Rosenwald et al. (2003) [13] determined that an increased level of expression of a set of 20 cell proliferation genes was a predictor of reduced survival of a sample of 92 patients diagnosed with MCL (LLMPP, http://llmpp.nih.gov). Rosenwald et al. (2001) [14] found a different 17 gene profile for predicting survival in DLBCL. These same data sets were obtained from LLMPP and re-analyzed for correlation of the AURKA (aurora A) and AURKB (aurora B) genes with MYC and BCL2 in MCL (n = 92) and DLBCL (n = 240) patients. Each probe was validated for annotation and probe values for each gene were averaged. Pearson correlation coefficients were calculated using R programming tools (http://www.r-project.org).

### Cell Proliferation Assay

Lymphoma cells were seeded at 10,000 per well in 96-well culture plates and allowed to grow for 24 hr followed by the desired treatment with increasing concentrations of the indicated agents (MLN8237, VCR) for 4 days. Viable cell densities were determined using a CellTiter 96 Cell Proliferation Assay (Promega). Absorbance readings at 490 nm were analyzed against the control group for each drug treatment to determine cell viability. The studies were performed in triplicates x 4 and IC_ 50_ values were estimated by Calcusyn software (Biosoft, UK). For combination studies of MLN8237 plus VCR, an equipotent ratio was calculated to determine a combined graded combination treatment. A control group was established for each drug treatment in six replicates. The effects of the combined treatments were determined by the combination-index (CI) and isobologram methods derived from the median-effect principle of Chou and Talalay.

### Cell Cycle Assay

Cells were treated with 50 nM of MLN8237 or rituximab 5 µg/mL or VCR 5 nM or combinations (M-R, VCR-R, M-VCR, M-VCR-R) for 24, 48 and 72 hr. Treated cells were centrifuged at 1,500×g for 5 min at 4°C and resuspended in PBS, fixed by drop-wise addition of ice-cold ethanol (100%) to a final concentration of 70%, and incubated for 30 min on ice. Fixed cells were pelleted and treated with 100 µl of RNase A (0.2 mg/ml in PBS) for 5 min at room temperature, then suspended in 1 ml ddH_2_O. After staining with 4 µg/ml propidium iodide, the DNA content was determined using a Becton Dickson flow cytometer and the cell cycle profile was analyzed by ModFit software. Cell aggregates were gated out of the analysis, based on the width of the propidium iodide fluorescence signal. Each profile was compiled from 10,000 gated events. All studies were performed in triplicate.

### Apoptosis Assay

Cells were treated with 50 nM of MLN8237 or rituximab 5 µg/mL or vincristine 5 nM or combinations (M-R, VCR-R, M-VCR, M-VCR-R) for 48 hr. Using Annexin V staining to detect apoptosis, treated cells were harvested and rinsed with cold PBS once. After centrifugation for 5 min, cells were resuspended in 500 µl of 1× Annexin V binding buffer (BioVision, Annexin V-FITC Reagent Kit, Cat.#1001-1000) and then added 5 µl of Annexin V-FITC and 5 µl of Propidium Iodide (BioVision, Annexin V-FITC Reagent Kit). After incubation for 5 min at room temperature in the dark, the samples were analyzed by flow cytometry. Apoptosis assessed by PARP-cleavage was conducted at the above concentration(s) of drug(s) for 48 hr.

### Immunoblotting

The cells were lysed in NP-40 lysis buffer containing 50 mM Tris.Cl (pH 7.4), 0.15 M NaCl, 0.5% NP-40, 1 mM DTT, 50 mM Sodium Fluoride, and 2 µl/ml Protease inhibitor cocktail (Sigma, St. Louis, MO). Protein concentrations were determined using the BioRad protein assay kit (Hercules, CA) and 50 µg of protein was resolved by electrophoresis on a 10% SDS-PAGE gel. The proteins were then transferred onto a nitrocellulose membrane and non-specific binding was blocked by incubating with 5% nonfat milk in TBST buffer (0.01 M Tris-Cl, 0.15 M NaCl, 0.5% Tween-20, pH 8.0) at room temperature for 1 hr. The membrane was subjected to the indicated antibodies and the proteins were detected by a LI-COR Odyssey Infrared Imaging System.

### MYC/BCL2 Co-expressing DLBCL Mouse Xenograft Model

Animal care and treatment were performed at Arizona Cancer Center's experimental mouse shared services (EMSS) core facility. SCID mice were injected with 1×10^7^ U-2932 DLBCL cells in matrigel subcutaneously into the right hind flank. When tumors reached a volume of ∼200 mm^3^, mice were divided randomly (pair-matched) into different groups with 12 mice per cohort. The mice were treated with MLN8237 [M, 30 mg/kg PO, QD for 3 weeks], rituximab [R, 10 mg/kg IV once/week, ×4], and vincristine [VCR, 0.375 mg/kg IV once/week, ×4] alone at indicated dosages or different combinations [M-R, M-VCR, VCR-R, and M-VCR-R]. The length (L) and width (W) of the subcutaneous tumors were measured by calipers and the tumor volume (T_V_) was calculated as: T_V_ = (L×W^2^)/2. Mice were sacrificed at the end of treatment (3 mice per cohort), end of study or if they reached >2000 mm^3^ at any time during the study. Excised tumors (end of the treatment) were either fixed in paraffin for immunohistochemistry (IHC) analysis or snap frozen for Western blotting and DNA microarray studies.

### RNA Isolation and Gene Expression Profiling

Total RNA was extracted from harvested tumors at the end of treatment (V-R versus M-VCR-R) using the RNeasy Mini Kit (Qiagen, CA). After checking for RNA quality, DNA microarray was performed utilizing the human HG-U133A Affymetrix (Santa Clara, CA) genechip consisting of 22 277 ‘probe sets’ (Genomic Core Facility at Arizona Cancer Center). Data analysis was performed using BioConductor libraries (http://www.bioconductor.org) and R programming (http://www.r-project.org). A quality control analysis for each array was performed using Affymetrix console methods and the affyQCReport library. The array data was background corrected and quantile normalized using the affy Bioconductor library. Probe set values for the same gene were summarized by averaging. To compare conditions, log ratios of expression values were calculated. For each comparison, the mean ±2 SD of the log fold changes were used as cut-offs to determine outlying differentially expressed genes. Each list of genes was tested for over-representation of KEGG and GO terms by counting the number of genes in the list with each pathway or term and using the Fisher exact test to determine over-representation. All terms with a *P*-value≤0.05 were reported.

### Statistical Analysis

All *in vitro* experiments were performed in triplicate. The data was expressed as mean ± S.D. The difference between two mean values were evaluated using the Student's *t*-test and considered to be statistically significant when *p*<0.05. Statistical analysis of the mouse xenograft model data was performed by estimating the tumor growth for each mouse by fitting the least squares regression line of the tumor volume by day. The cube root of the observed tumor volumes was used to induce linearity in the raw data values. The slope of the regression line measures the tumor growth rate. Analysis of variance was used to test for the overall treatment effects on TGI. Tukey's studentized range test was used to assess the significance of pair-wise differences between the groups adjusted for multiple comparisons. Survival of the mice was measured from the date of pair matching to sacrifice (event) or end of study (censored). The Kaplan-Meier method was used to estimate survival. The log rank test was used to compare survival between the respective treatment groups. Statistical adjustments were made for multiple comparisons. Analysis was performed using Prism (Graphpad, La Jolla, CA). All p-values≤0.05 were considered statistically significant.

## Results

### Aurora A and B Expression Correlates with MYC and BCL2 Expression in B-NHL

Previously we analyzed the effect of expression of AURKA (aurora A) and AURKB (aurora B) genes on MCL patient survival (LLMPP, http://llmpp.nih.gov) [12], [13] and showed both were predictive markers in MCL. For DLBCL [14] the Kaplan-Meier survival curves did not correlate with Aurora A or B expression (data not shown) but a tissue microarray confirmed both aurora genes are over-expressed in DLBCL (data not shown) implying that they remain excellent therapeutic targets. We re-analyzed the LLMPP data [MCL (n = 92) and DLBCL (n = 240)] (13, 14) for correlative expression of AukA, AukB, MYC and BCL2 with respect to each other to ascertain potential functional interactions (**Table 1**). AukA and AukB are highly correlated in both MCL and DLBCL. MYC demonstrated a strong correlation with AukA and AukB in MCL and weakly correlated with AukA and AukB in DLBCL. BCL2 moderately but significantly correlated with AukA and AukB in DLBCL but not in MCL. However, MYC and BCL2 did not correlate in either data set. These data provide evidence that Aurora A and B expression intra-correlate and inter-correlate with MYC and BCL2 expression in MCL and DLBCL setting the stage for *in vitro* and *in vivo* investigation.

**Table 1:** Correlating the expression of Aurora A and B with MYC and BCL2 in MCL and DLBCL [LLMPP database].

Pearson Correlation Coefficients, Mantle Cell Lymphoma			
Gene	AURKA	AURKB	BCL2
AURKA	-	0.80	0.10*
MYC	0.58	0.49	-0.14*
BCL2	0.10*	−0.10*	-

Pearson Correlation Coefficients, Diffuse Large B-NHL			
Gene	AURKA	AURKB	BCL2
AURKA	-	0.76	0.41
MYC	0.27	0.24	0.02*
BCL2	0.41	0.26	-

*Not significant P>0.3.

### MYC, BCL-2 and p53 are Differentially Expressed in Aggressive DLBCL Cell Lines

**Figure 1:**
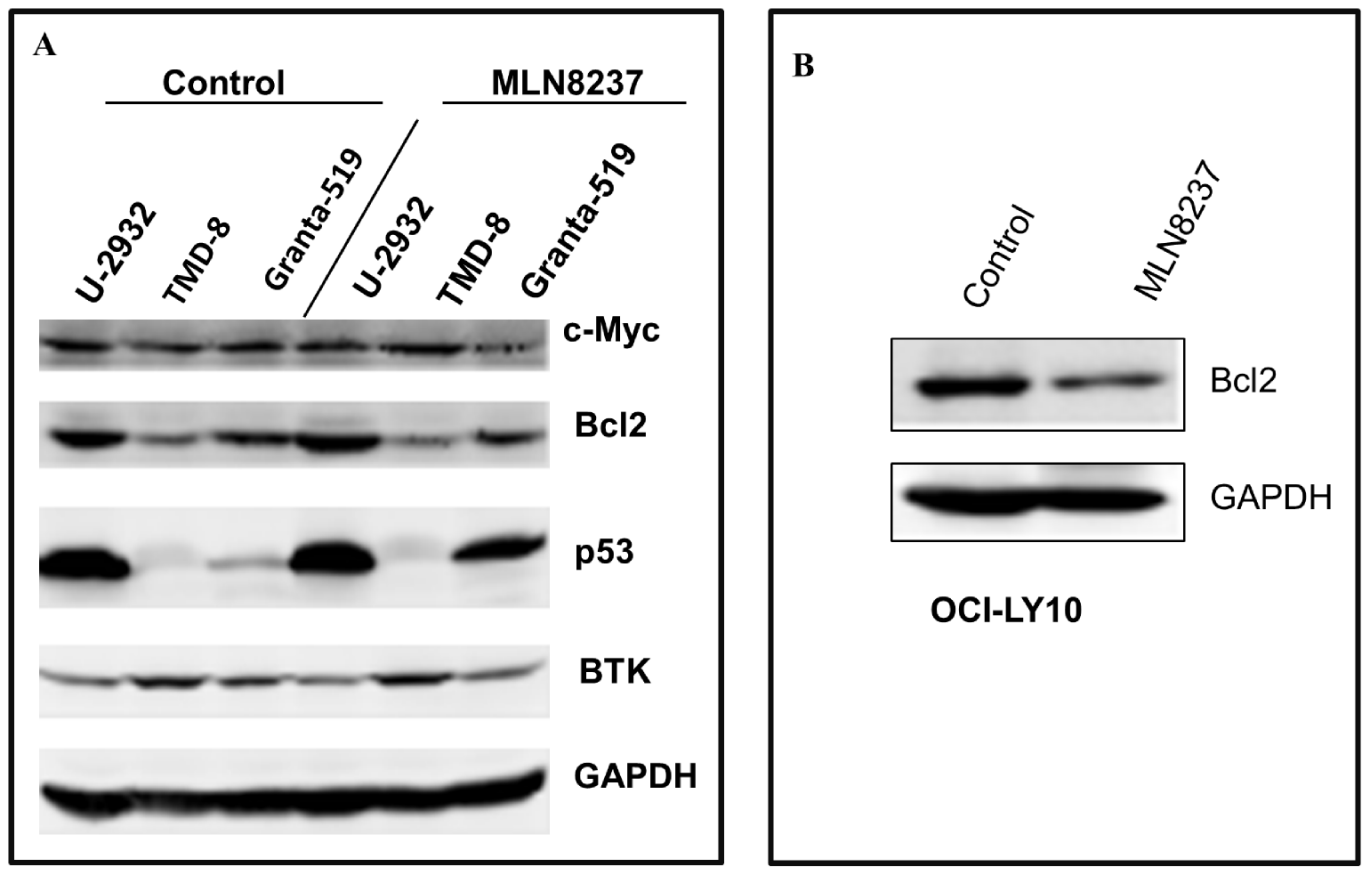
Differential expression of MYC, BCL-2 and p53 protein levels in aggressive DLBCL cell lines. (**A**) Western blotting analysis of concurrent c-Myc & Bcl-2 protein expression in U-2932, TMD-8, and Granta-519 cell lines in the absence and presence of MLN8237 (1.5 µM for 48 hr). The levels of p53 and BTK expression are evaluated + MLN8237. (**B**) Western blotting analysis of Bcl-2 protein expression in OCI-Ly10 cell line in the absence and presence of MLN8237 (1.5 µM for 48 hr).

The objective of the study was to evaluate differential expression of Myc, Bcl-2 and p53 in the ABC-DLBCL cell lines U-2932, TMD-8, OCI-Ly10 and MCL cell line Granta-519 (**Table 2**). Western blotting analysis demonstrated that c-Myc to be equally expressed in U-2932, TMD-8 and Granta-519 cell lines but not in OCI-Ly10 (**Figure 1**). In contrast, Bcl-2 is highly expressed in U-2932, and with moderate to low expression in OCI-Ly10, Granta-519 and TMD-8 cells, respectively (**Figure 1**). P53 is highly over-expressed in U-2932, with no expression in TMD-8 and OCI-Ly10. BTK is over-expressed in TMD-8 cells, in contrast to U-2932 and Granta-519 cells where moderate expression is observed. Together, the data indicate concurrent differential Myc and Bcl-2 expression in DLBCL and MCL cell lines.

**Table 2:** Aggressive B-NHL cell line characteristics.

B-NHL Cell Line	Genotype
Granta-519	Cyclin D1 [t (11;14)], ATM (HI), p16/p14 (Del), p53 (wt) c-Myc (OE), Bcl2 (OE)
U2932	Amp 18q21 [Bcl6], Amp 3q27 [Bcl2-OE], p53 (m-OE), c-Myc (OE) CARD 11 (WT), BCR/NF-ĸB Active
TMD8	CARD 11 (WT), CD79B (ITAM Y 196H) BCR/NF-ĸB Active, NOTCH1, p53 (wt) c-Myc (OE), Bcl2 (OE)
OCI-LY10	CARD 11 (WT), CD79B (ITAM del), MYD88 mutation, BCR/NF-ĸB Active, p53 (wt)

### Synergistic Interaction between a Microtubule Depolymerizer and Aurora Inhibition [alisertib, MLN8237] in DLBCL Cell Lines

**Figure 2:**
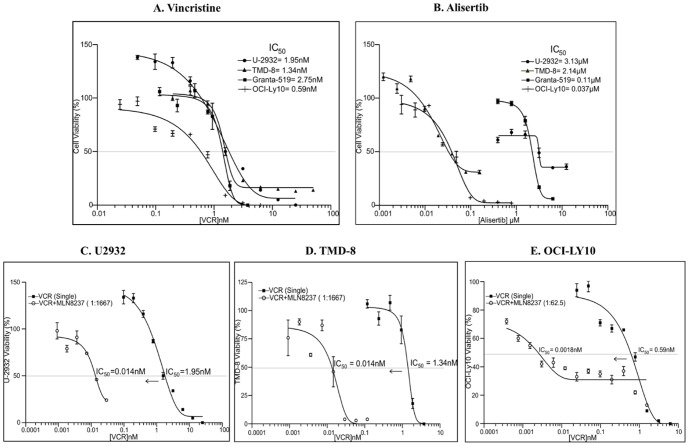
Combination of alisertib plus vincristine is highly synergistic in DLBCL cells. Cell proliferation (MTS) assays of U-2932, TMD-8, OCI-Ly10 and Granta-519 cells treated with MLN8237 [M] or Vincristine [VCR] or M-VCR. (**A and B**). The IC_50_'s for the B-NHL cell lines treated with single agents are: U-2932 [M = 3.1 µM, VCR = 1.95 nM], TMD-8 [M = 2.1 µM, VCR = 1.34 nM], OCI-Ly10 [M = 37 nM, VCR = 0.59 nM] and Granta-519 [M = 110 nM, VCR = 2.75 nM]. (**C, D and E**). For the combination, the dose ratio was 1∶1667 (VCR:M) for U-2932 and TMD-8 or 1∶62.5 (VCR:M) for OCI-Ly-10 cell line. All DLBCL cell lines experienced a strong synergism with M-VCR therapy.

When these B-NHL cell lines are treated with alisertib (MLN8237) at 1.5 µM for 48 hr, the level of expression c-Myc, BTK and GAPDH does not change, but Bcl-2 is downregulated in OCI-Ly10 and p53 is upregulated in the MCL Granta-519 cells (**Figure 1**) indicating activation of the G0/G1 checkpoint. The goal of the study was to investigate synergy of interaction between microtubule disassembly dynamics and Aurora inhibition. First, B-NHL cells (U-2932, TMD-8, OCI-Ly10) were treated in serial dilution with MLN8237 or vincristine. The IC_50_'s for the B-NHL cell lines treated with single agents are: U-2932 [M = 3.1 µM, V = 1.95 nM], TMD-8 [M = 2.1 µM, V = 1.34 nM] and OCI-Ly10 [M = 37 nM, V = 0.59 nM]. For the combination, the dosage ratio was approximately 1∶1667 (V:M) for U-2932 and TMD-8 and 1∶62.5 for OCI-Ly10 cells. All 3 DLBCL cell lines experienced a synergistic response with the combination of MLN8237 and Vincristine. The CI values for IC_50_s were determined to be 0.02 for U-2932 and TMD-8 and 0.0062 for OCI-Ly10 cells respectively indicating exquisite synergy of interaction (**Figure 2** **,** **Table 3**).

**Table 3:** Combination indices derived from the median-effect principle of Chou and Talalay for M-VCR combination in aggressive B-NHL cell lines.

B-NHL Cell Line	Combination Index (CI)
U2932	0.02±0.001
TMD8	0.02±0.002
Granta-519	0.05±0.008
OCI-LY10	0.0062±0.001
	<0.1 Very Strong Synergism

### Alisertib Plus Vincristine plus Rituximab Overcomes Cell Cycle Checkpoints and Induces Apoptosis in DLBCL Cells

**Figure 3:**
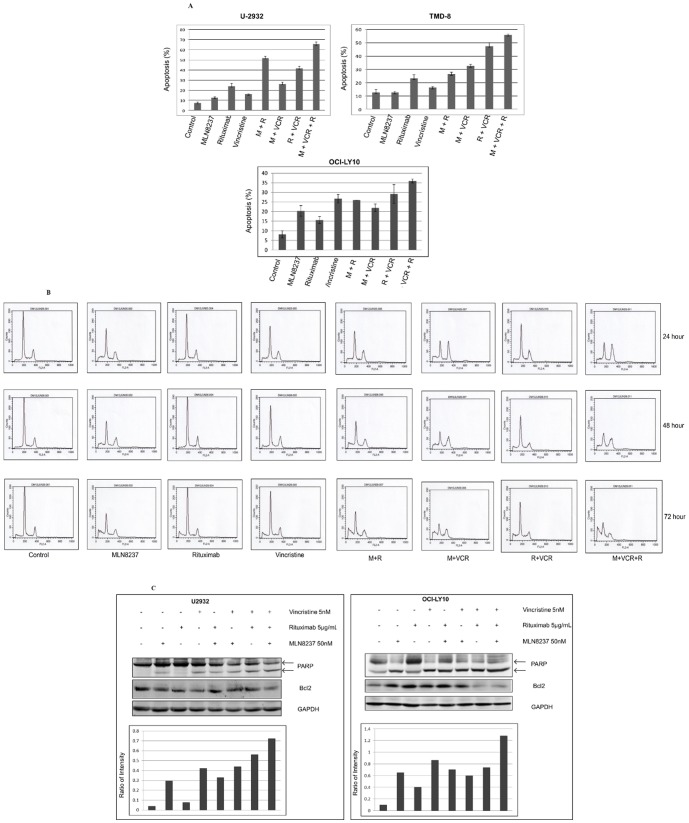
Alisertib added to Vincristine plus Rituximab overcomes cell cycle checkpoints and induces apoptosis in DLBCL cells. (**A**) U-2932, TMD-8 and OCI-Ly10 cells were treated with VCR = 5 nM, R = 5 µg/ml and MLN8237 = 50 nM alone or the combinations as indicated at same doses for 48 hr. Apoptosis was analyzed by flow cytometry after annexin V and PI staining. The graph represents the mean percentage of apoptosis ± S.D. (n = 3). (**B**) U-2932 cells were treated as same as (**A**) for 24hr, 48 hr and 72 hr, DNA profiles were evaluated by flow cytometry after PI staining. (**C**). U-2932 and OCI-Ly10 cells were treated as same as (A) for 48 hr, PARP-cleavage and Bcl2 levels were analyzed by Western blotting. The band intensity of cleaved PARP was quantitated using Western blot analysis software (Li-Cor), normalized to GAPDH and graphed.

Rituximab [R] is an effective treatment when combined with chemotherapy in B-NHL. To determine whether R enhances cytotoxicity of M or VCR and doublet combinations, we evaluated degree of apoptosis by flow cytometry in U-2932, TMD-8 and OCI-Ly10 cell lines utilizing sub-lethal doses (VCR: 5 nM, R: 5 µg/ml, M: 50 nM) treated for 48 hr. As shown in **Figure 3**, R is more effective in inducing apoptosis that M or VCR compared to control in U-2932 and TMD-8 cells. For the combination treatments, M-R>M-VCR and VCR-R [U-2932 cells], and VCR-R>M-VCR and M-R [TMD-8 and OCI-Ly10 cells] significantly induced apoptosis compared to single agents and control. Importantly, M-VCR-R triple therapy significantly enhanced apoptosis over any of the doublets (**Figure 3A**). Cell cycle analysis of B-NHL cells [U-2932] treated with sub-lethal doses of drug(s) (VCR: 5 nM, R: 5 µg/ml, M: 50 nM) as single or combinations at 24 hr, 48 hr and 72 hr demonstrate that M-VCR-R is effective in overcoming the G0/G1 and G2/M checkpoints and induce apoptosis compared to single or doublet therapies (**Figure 3B**). Apoptosis by PARP-cleavage utilizing the above drug doses with B-NHL cells treated for 48 hr indicates M-VCR-R is effective in inducing cell death compared to single or doublet therapies. Further, Bcl2 levels also decrease significantly with increased PARP cleavage with M-VCR-R therapy (**Figure 3C**).

### Alisertib Added to Rituximab Plus Vincristine Appears to be Active in a MYC/BCL-2 Co-expressing DLBCL Mouse Model

**Figure 4:**
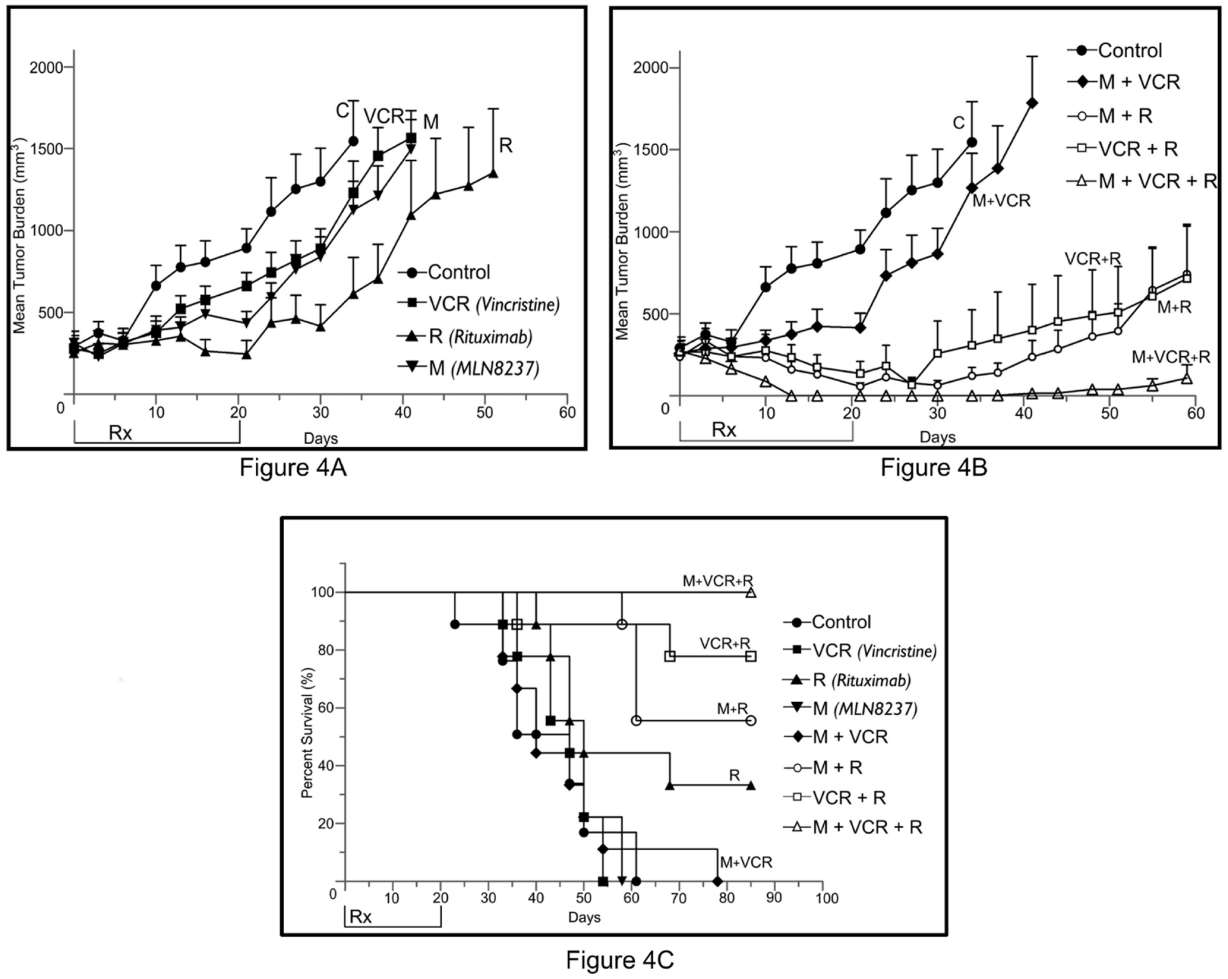
MLN8237 plus Vincristine plus Rituximab is synthetic lethal in a mouse xenograft model of DHL. (**A**). U-2932 xenograft mice (n = 12 per cohort) were treated with saline (control), M = 30 mg/kg, R = 10 mg/kg and VCR = 0.375 mg/kg. MLN8237 was given by PO Q1D ×3 weeks, VCR and rituximab by IV Q1W ×4 weeks. (**B**). U-2932 xenograft mice (n = 12 per cohort) were treated with M-VCR, M-R, R-VCR and M-VCR-R with the above doses and schedules. For (**A**) and (**B**) the tumor burden was measured, graphed and represented as mean ± S.E.M. (**C**). Kaplan-Meier survival curves show overall survival differences between M-VCR-R in comparison to control, M, R, VCR, M-VCR, M-R and VCR-R. (**D**). Western blotting analysis of tumors harvested from VCR-R and M-VCR-R shows modulation of karyokinesis regulating genes CENP-C and Aurora B.

Based on *in vitro* data of inhibition of cell proliferation and abrogation of cell cycle checkpoints with associated enhanced apoptosis, we evaluated the effectiveness of M-VCR-R in Myc+/Bcl2+U-2932 SCID mouse xenograft model. There were 9 cohorts of 12 mice: vehicle control, MLN8237 [M, alisertib] at 30 mg/kg PO once a day for 3 weeks, R at 10 mg/kg IV once/week × 4, VCR 0.375 mg/kg IV, once a week ×4, doublets [M-VCR, VCR-R, M-R] and M-VCR-R administered at the above clinically utilized doses. Treatments with M or VCR alone showed a ∼10-15% TGI compared to vehicle control. However, R showed significant TGI of ∼50% compared to vehicle control (p<0.05) (**Figure 4A**) with an OS (Kaplan-Meier) of 20 days beyond the control arm (p<0.05) (**Figure 4C**).

Treatments with the doublets demonstrated that M-R = VCR-R>>M-VCR, with tumor regression [TR] observed with M-R and VCR-R. However, after initial tumor regression, M-R and VCR-R treated tumors relapsed 10 days after end of treatment. Treatment with M-VCR was mostly stable disease followed by tumor growth with a TGI of ∼10-15% similar to that observed with single agent M or VCR (**Figure 4B**). Tumor response to M-R and VCR-R are reflected in the Kaplan-Meier OS curve which indicates >50% mice are alive 65 days after end of treatment compared to M-VCR where most mice are dead 35 days after end of treatment (p<0.01) (**Figure 4C**).

Treatment with M-VCR-R led to tumor regression compared to control or single agents or doublets (p<0.001 and p<0.001), respectively (**Figure 4B**). Interestingly, 3 of 9 tumors treated with M-VCR-R relapsed 30 days after end of treatment. Kaplan-Meier analysis of OS showed that the mice treated with M-VCR-R had a statistically significant improvement in overall survival when compared with the control (p<0.0001) or single agents or doublets (p = 0.0043) (**Figure 4C**). At the end of the study which was >120 days after end of treatment 6 mice were tumor free. The mice in all cohorts tolerated treatment well with no weight loss >10%.

### M-VCR-R Modulates Centromere Associated Genes in Mitotic Survival

Tumors were harvested at end of treatment (3 weeks) from control, VCR-R and M-VCR-R cohorts for gene expression profiling to ascertain which genes were changing with the addition of MLN8237 to VCR-R, that influenced tumor relapse observed with VCR-R. The mice were carefully observed during VCR-R and M-VCR-R treatment due to rapid tumor regression. Of the 12 mice in each cohort, 3 mice had palpable tumors toward the end of treatment (harvested), while the remaining 9 mice had barely or no palpable tumors. GO identified 18 up-regulated genes represented within the centromere-kinetochore complex important for regulating karyokinesis during the mitotic phase of the cell cycle (Fisher Exact Test, p<0.05). RT-PCR analysis of SGOL-2, CENP-e (kinesin-7) and KIF20A showed 2-fold changes indicating continued regulation of karyokinesis modulating genes that promote a disruptive mitosis with M-VCR-R therapy however is an escape mechanism with R-VCR therapy, as all tumors relapse in this treatment arm (Table S1). Western blotting analysis indicated CENP-C is up-regulated while Aurora B is down-regulated with M-VCR-R versus VCR-R respectively (**Figure 4D**), implicating continued mitosis in the presence of synthetic lethality.

## Discussion

*MYC*-driven double hit diffuse large B-cell lymphoma (DHL) is a molecularly defined subset with an aggressive clinical course that requires novel targeted combinations of agents to provide a high impact on survival. There is no data from prospective trials that specifically address *MYC+/BCL2*+DHL patients but retrospective subset analyses indicate that patient with DHL do poorly when treated with *R*-CHOP [4], [15] with a 5-year overall survival of 33% with *MYC* rearrangement compared to 72% of patients who lacked MYC rearrangements. Treatment failures during or after primary treatment of DHL are rarely salvaged with R-ICE or R-DHAP followed by high-dose BEAM and auto stem-cell transplantation [16]. In the relapsed and refractory setting, salvage chemotherapy and auto stem-cell transplantation outcomes are extremely poor for patients with *MYC*+ disease (CORAL study: Collaborate Trial in Relapsed Aggressive Lymphoma), where 75% patients had FISH evidence of DHL. Regimens [CODOX-M/IVAC] thought to be active in Burkitt lymphoma (*MYC* translocation) did not confirm effectiveness [17]. A possible treatment for *MYC*+DLBCL is dose-adjusted R-EPOCH [18], however, *MYC*+ patient numbers are limited and BCL2 status is unknown, hence no conclusions can be drawn.

We **hypothesized** that co-expression of Myc and Bcl2 is functionally equivalent to translocation seen in DHL targetable by inhibition of Aurora in the presence of MTAs (e.g. VCR) plus rituximab. A re-analysis of the LLMPP data [MCL (n = 92) (13)] and DLBCL (n = 240)] (14) for correlative expression of Aurora A, Aurora B, MYC and BCL2 with respect to each (**Table 1**) demonstrated that Aurora A and B expression is highly correlated in both MCL and DLBCL. MYC expression demonstrated a strong correlation with Aurora A and B in MCL but moderately correlated with Aurora A and B in DLBCL. BCL2 expression moderately but significantly correlated with Aurora A and B in DLBCL but not in MCL. In contrast, MYC and BCL2 expression did not correlate in either DLBCL or MCL.

The status of mutated or wild type p53 operating within the chronic active BCR pathway may also be relevant to DHL drug resistance. We demonstrate that U-2932, TMD-8, and Granta-519 cells (**Table 2**) co-express Myc and Bcl2 within an active BCR signaling pathway (**Figure 1**). Treatment with alisertib up-regulated wild type p53 in Granta-519 MCL cells indicating G0/G1 arrest consequent to G2/M checkpoint activation. This effect was not observed in U-2932 DLBCL cells where p53 is mutated (**Figure 1**) implicating a differential response to alisertib. In cell proliferation assays, Granta-519 and OCI-Ly10 cells have similar IC_50_s in the range of 37 to 110 nM compared to U-2932 and TMD-8I with IC_50_s from 2.14 to 3.13 µM (**Figure 2**). Vincristine [VCR] is active with an IC_50_ of 0.59 to 2.75 nM in all DLBCL cells and the combination with alisertib [M] was highly synergistic (**Figure 2** **,** **Table 3**). Mechanistic synergy of M-VCR in MCL cells [9] implicates synthetic lethality in aggressive B-NHL in cell culture irrespective of p53 and BCR status.

Apoptosis assays by flow cytometry confirm enhanced activity of M-VCR versus M or VCR (**Figure 3A**). Cell cycle analyses indicate M-VCR-R is the most potent combination in causing loss of G0/G1, G2/M arrest, overriding the spindle checkpoint leading to increased apoptosis in a time-dependent manner (**Figure 3B**). Finally, M-VCR-R is the most effective combination that causes PARP cleavage with >50% loss of Bcl2 expression (**Figure 3C**) which corroborates well with loss cell cycle checkpoints and subsequent apoptosis (**Figure 3A and 3B**).

In a U-2932 DLBCL mouse model rituximab [R] was superior to M, VCR or M-VCR in causing TGI with associated superior OS. Despite the excellent synergy observed with M-VCR in DLBCL and MCL in cell culture and tumor regression observed in a mouse Granta-519 model [9], M-VCR is inferior in the U-2932 DLBCL model. The doublets M-R and VCR-R were superior to M-VCR in DLBCL apoptosis assays (**Figure 3A**) and corroborates well with anti-B-NHL activity in the DLBCL mouse model. However, M-VCR-R caused tumor regression in both the Granta-519 [9] and U-2932 mouse models with 100% overall survival (**Figure 4B and 4C**). Hence, addition of R to M-VCR (Granta-519) or M to VCR-R (U-2932) is synthetic lethal and curative of both types of aggressive B-NHL. Although M-VCR is not active in the U-2932 mouse model, addition of R overcomes the mutated p53 driven Myc+/Bcl2+ co-over-expression. Gene expression profiling identified 18 up-regulated genes when comparing M added VCR-R *versus* VCR-R. These 18 up-regulated genes are predominantly associated with associated karyokinesis. Modulation of CENP-C and Aurora B proteins in response to M-VCR-R therapy can now be evaluated in prospective clinical trials as potential biomarkers of response and/or resistance to therapy.

In conclusion, collectively our data suggest that the interaction between alisertib plus VCR plus rituximab is synergistic and synthetic lethal in DLBCL co-expressing Myc and Bcl2. Alisertib [M] plus vincristine plus rituximab [M-VCR-R] has completed an early phase trial evaluation in relapsed and refractory aggressive B-NHL (C14011) [ClinicalTrials.gov].

## Supporting Information

Table S1 **Genes up-regulated with M-VCR-R versus R-VCR treatment.**
(TIF)
