# Supplementary figures and images for: Alisertib Added to Rituximab and Vincristine Is Synthetic Lethal and Potentially Curative in Mice with Aggressive DLBCL Co-Overexpressing MYC and BCL2
Source: PLoS One. 2014 Jun 3;9(6):e95184. doi: 10.1371/journal.pone.0095184 (PMC4043492; doi:10.1371/journal.pone.0095184)

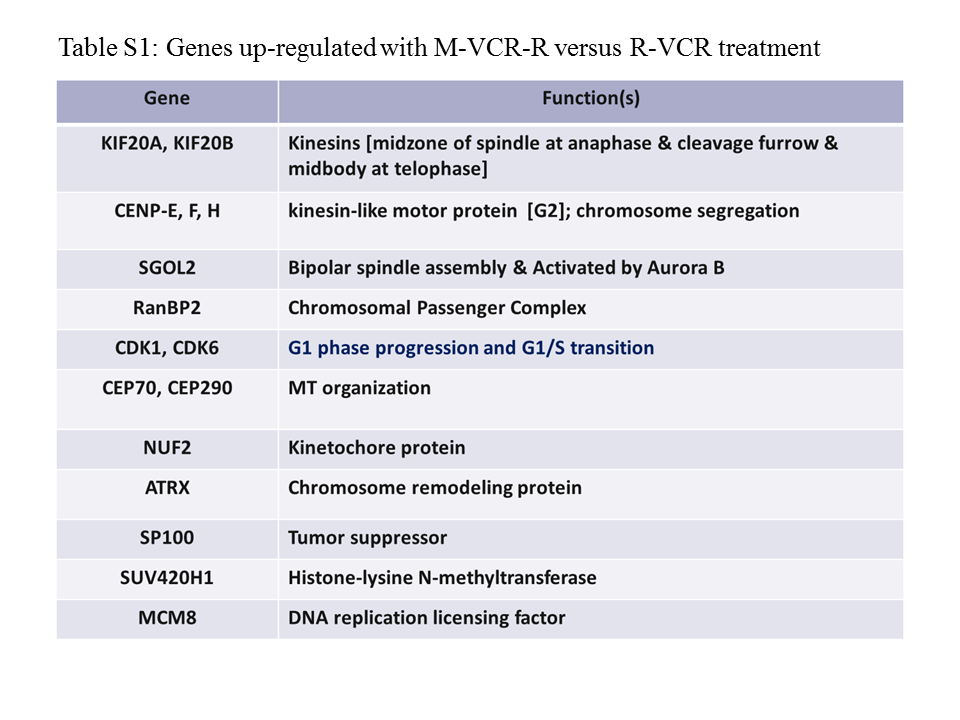

Supplement: Table S1 — Genes up-regulated with M-VCR-R versus R-VCR treatment. (TIF) [file pone.0095184.s001.tif]
